# Neural Activity Disparities in Deficiency and Excess Patterns of Depression: Protocol for a Systematic Review and Meta-Analysis

**DOI:** 10.2196/68996

**Published:** 2025-09-18

**Authors:** Liling Chen, Xinyu Jia, Jianjun Wang, Liling Li, Jianxiang Chen, Songjun Lin, Haotao Zheng, Xinbei Li, Xiude Qin, Lanying Liu, Hanqing Lyu

**Affiliations:** 1 The Fourth Clinical Medical College Shenzhen Traditional Chinese Medicine Hospital Guangzhou University of Chinese Medicine Shenzhen China; 2 Department of Radiology Shenzhen Traditional Chinese Medicine Hospital Shenzhen China; 3 Department of Neurology and Psychology Shenzhen Traditional Chinese Medicine Hospital Shenzhen China; 4 Shanghai Mental Health Center Shanghai Jiao Tong University School of Medicine Shanghai China; 5 Shanghai Institute of Traditional Chinese Medicine for Mental Disorders Shanghai China

**Keywords:** depression, Chinese Medicine, functional magnetic resonance imaging, fALFF, fractional amplitude of low-frequency fluctuations, subtype

## Abstract

**Background:**

Major depressive disorder (MDD) is a complex and heterogeneous condition. Current diagnosis relies on symptom-based assessments, leading to varied treatment responses. Data-driven approaches have attempted to identify MDD subtypes, but their clinical applicability remains limited. Traditional Chinese Medicine (TCM) provides a theory-driven classification system that categorizes MDD into syndrome subtypes of deficiency pattern and excess pattern, offering insights into the biological mechanisms and personalized treatment strategies.

**Objective:**

This systematic review and meta-analysis aims to identify potential neurobiological distinctions of TCM-based deficiency and excess patterns in MDD by examining differences in the brain activity by using various functional magnetic resonance imaging (fMRI) modalities, including resting-state and task-based fMRI, diffusion tensor imaging, and magnetic resonance spectroscopy.

**Methods:**

In accordance with the Preferred Reporting Items for Systematic Reviews and Meta-Analyses Protocols (PRISMA-P), we will conduct a comprehensive search of 7 electronic databases (PubMed, Embase, Web of Science, Chinese Biological Medical Literature database, China National Knowledge Infrastructure, Chinese Wanfang database, and Chongqing VIP database) for studies published up to December 2024. Eligible studies will be screened by 2 independent reviewers based on predefined inclusion criteria, followed by data extraction and quality assessment. For the meta-analysis, resting-state fMRI studies will be analyzed in Montreal Neurological Institute space using Seed-based d Mapping-Permutation of Subject Images software (version 6.21), enabling a focused evaluation of brain activity differences in deficiency and excess MDD patterns.

**Results:**

The search and screening for the systematic literature review were completed in December 2024. This study relies on published, publicly accessible data. We found approximately 30 eligible studies in our preliminary search, suggesting that a quantitative meta-analysis is feasible. Data extraction, quality appraisal, and subsequent data synthesis will begin in September 2025. The review should be completed by December 2025, and the study results will be published in 2026.

**Conclusions:**

The results of this study may help to explain the neural mechanisms of depression’s neurobiological subtypes from the perspective of TCM.

**Trial Registration:**

PROSPERO CRD42023475178; https://www.crd.york.ac.uk/PROSPERO/view/CRD42023475178

**International Registered Report Identifier (IRRID):**

DERR1-10.2196/68996

## Introduction

Major depressive disorder (MDD) is a complicated disease that imposes a significant worldwide burden of disease [1]. Currently, the diagnosis of MDD depends mainly on structured interviews and scale scores that measure symptoms, functions, and personality [1,2]. This approach leads to significant confusion in the classification of MDD. The treatment response of MDD differs among different patient populations due to factors such as the lack of specificity in symptoms, along with individual variations in the intensity and duration of the disease [3-5]. Therefore, researchers are devoted to investigating the specific etiology and pathology of MDD and identifying more homogeneous subgroups [6]. The existing classification paradigm for MDD subgroups consists of 3 main categories: neurobiological mechanisms [7], genetic variants [8-10] and symptom patterns, as well as clinical characteristics [11,12]. Researchers apply data-driven methods to identify patterns of different subtypes [13,14], with the goal of understanding their clinical significance in treatment responses and predicting individual variations in cognitive and emotional profiles [15-17]. However, the accuracy of these classification outcomes is seldom duplicated in separate test samples, thereby restricting their utility in actual clinical environments [15,18].

Interestingly, in contrast to the data-driven approaches, the syndrome-based classification model of Traditional Chinese Medicine (TCM) is a dependable system for classifying medical conditions, and it has practical use in clinical settings. The TCM syndrome comprises different symptoms and signs that represent the fundamental characteristics of a specific stage of a disease. Syndrome differentiation in TCM is a concise summary of the pathological changes underlying the clinical manifestations. It is determined by the patient’s metabolic features and susceptibility to disease and serves as the foundation for clinical practice in TCM [19]. In particular, the clinical value of this classification model has been widely proven in thousands of years of practice. However, this framework for classifying subtypes, which is rooted in the TCM theory, requires a deeper investigation into the biological mechanisms underlying their occurrence. It is crucial to identify objective indicators based on the biological substrates associated with different syndrome types [20]. This will enhance the efficiency of TCM diagnosis and promote the development of standardized diagnostic indicators for MDD.

The TCM syndrome subtypes are assessed using 4 diagnostic methods: inspection, listening/smelling, inquiry, and palpation. Following the classification and codes of diseases and patterns of TCM (GB/*t* 15657-2021), the 2 most commonly applied classifications for MDD in TCM are deficiency pattern (DP) and excess pattern (EP). DP is characterized by clinical manifestations such as fatigue, low energy, pale tongue, and weak pulse, indicating a lack of qi, blood, or organ function. EP is manifested by symptoms such as irritability, chest tightness, constipation, a red tongue with yellow coating, and a wiry pulse, suggesting internal heat, stagnation, or phlegm accumulation.

Currently, functional magnetic resonance imaging (fMRI) offers a nonintrusive method for examining the structure and function of the brain [21]. Despite being more costly and difficult to obtain, various fMRI sequences can detect differences in brain activations and microstructural changes in groups, including those previously thought to be “normal” like depression by using quantitative analyses instead of visual assessments [22]. The general fMRI sequences include blood-oxygen-level–dependent imaging, diffusion MRI, and magnetic resonance spectroscopy. Various studies have employed these techniques and reported that individuals with MDD exhibit regional neural activity disorders, abnormalities in functional connectivity within and between brain areas, and changes in brain structures such as white matter [23-25]. Moreover, multiple fMRI studies on TCM syndrome subtypes have demonstrated distinct fMRI activation patterns in different TCM syndrome subtypes of MDD, highlighting distinct brain responses among these subtypes [26-30]. Further, the high spatial resolution of whole-brain fMRI is in line with TCM’s holistic diagnostic approach, enabling a comprehensive mapping of brain responses corresponding to different TCM syndromes in depression.

Hence, we aim to conduct a comprehensive literature review to elucidate the neural mechanisms underlying the neurobiological subtypes of depression through the lens of Chinese Medicine syndromes. This will involve a combined use of quantitative and qualitative methodologies. We will focus particularly on studies that classify patients into DP or EP subtypes by using standardized syndrome differentiations based on TCM guidelines.

## Methods

### Study Registration

We performed our protocol in accordance with PRISMA-P (Preferred Reporting Items for Systematic Reviews and Meta-Analyses Protocols) guidelines [31] (Multimedia Appendix 1). This study was registered with PROSPERO (CRD42023475178).

### Eligibility Criteria

Studies using fMRI to investigate the brain mechanism of depression based on TCM syndromes are included. All TCM syndrome subtypes must follow the classification and codes of diseases and patterns of TCM (GB/*t* 15657-2021) and be classified into DP and EP. Depression is diagnosed based on the standard diagnostic criteria, including the Diagnostic and Statistical Manual of Mental Disorders, the International Classification of Diseases, and the Chinese Classification of Mental Disorders. TCM syndrome differentiation follows the criteria for the diagnosis and the therapeutic effects of diseases and syndromes in TCM. Studies that meet the following criteria are included in the meta-analysis: (1) used an fMRI voxel-wise whole-brain approach of resting-state activation data; (2) reported altered spontaneous functional activities in local brain regions; and (3) reported brain regions with abnormal activations as x/y/z coordinates in standard space: Montreal Neurological Institute template or Talairach atlas.

The following types of studies are excluded: (1) reviews/meta-analyses/single-case studies/dissertations/conference papers/abstracts; (2) letter/comments/viewpoints/summaries for patients/editorials and opinion papers; (3) no assessment of TCM syndromes as exposure; (4) exposure to any active TCM interventions and non-TCM ones such as acupuncture, herbal medicine, moxibustion, massage, psychotherapy, pharmacological treatments, or brain stimulation techniques; (5) animal studies; and (6) duplicate publications of the same data.

### Search Strategy

#### Data Sources

Two independent reviewers (LC and XJ) systematically searched for literature up to December 2024 by using PubMed, Embase, Web of Science, Chinese Biological Medical Literature database, Chinese National Knowledge Infrastructure database, and the Chinese Wanfang and Chongqing VIP databases. The search was conducted using a combination of Medical Subject Headings (MeSH) and unrestricted terms related to (1) depression, (2) Chinese medicine, and (3) MRI.

#### Search Terms

No limitations will be placed on the publication’s time or location. To make sure the search includes the latest data, it is intended to be updated before the final review is submitted. The terms that were used in the search strategy are shown in Multimedia Appendix 2.

### Data Management

Every study obtained from the search will be loaded into EndNote, and any duplicates will be eliminated. Two authors (LC and XJ) will independently review the titles, abstracts, and keywords of these studies based on the predefined criteria for inclusion and exclusion. Following that, the studies selected will undergo a second screening process by thorough reading of the complete text. Studies that do not match the specified review criteria will be excluded. If there is a disagreement, a third author (HL) will be responsible for making the ultimate decision.

### Data Extraction

Data extraction will also be performed by 2 independent reviewers (LC and XJ). We will design a structured data extraction form based on the fMRI study reporting criteria in order to ensure standardization in the process of extracting data. Any ambiguity or disagreement will be resolved by consulting a third investigator (HL). The following data will be extracted: first author, publication year, study type, sample size per group, age, gender, drug history, diagnosis criteria, ratings of depression and anxiety, modality and metrics of fMRI, type of task performed during functional imaging, fMRI device type, sequence parameters, software for fMRI data analysis, quality score, and reported results. Moreover, the significance level and the peak coordinates with its 2-sided *t* value or *z* score or *P* value will be extracted for the meta-analysis.

### Assessment of Study Quality

We will use the Agency for Healthcare Research and Quality scale to evaluate the quality of the clinical research based on the specific types of studies included [32]. Additionally, an fMRI checklist will be used to access the imaging methodology quality. This checklist involves the inclusion and exclusion criteria, population characteristics, diagnosis criteria, sample size, methods for image acquisition and analysis, and the quality of reporting of statistical results and research conclusions [33]. Similarly, the evaluation of quality will be conducted independently by 2 reviewers (LC and XJ). A third researcher (HL) will resolve any disagreement that arises during the evaluation procedure. We will categorize the literature into high, medium, and low quality according to their scores.

### Strategy for Data Synthesis

The synthesis of data from the selected studies will involve a combination of quantitative and qualitative methods. We will systematically summarize the clinical and methodological characteristics of the studies through tables and narratives, detailing imaging techniques, statistical approaches, TCM syndromes, and fMRI biological substrates associated with each syndrome. To improve data clarity, we concentrate on the primary DP and EP within the framework of syndrome subtypes methodology as applied in TCM clinical practice. The qualitative synthesis will follow a thematic content analysis approach, using a structured data extraction matrix to categorize imaging findings by brain region, activation direction (increased/decreased), and syndrome type (DP vs EP). Findings will be coded and grouped into emergent neurofunctional patterns that characterize each TCM subtype. The quantitative investigation will assess and contrast the neuroimaging characteristics among the 3 subgroups. We will also perform a direct comparison between the DP and EP groups to identify specific neural activation differences associated with TCM-defined subtype distinctions. Three sets of comparisons will be conducted in the meta-analysis: (1) DP versus healthy control (HC), (2) EP versus HC, and (3) DP versus EP.

The meta-analysis of resting-state fMRI studies will be conducted in Montreal Neurological Institute space using Seed-based d Mapping-Permutation of subject images software (version 6.21). For each comparison (DP vs HC, EP vs HC, and DP vs EP), we will generate effect size maps based on reported peak coordinates and statistical values. Quantitative outcomes of interest will include fractional amplitude of low-frequency fluctuations, amplitude of low-frequency fluctuations, regional homogeneity, and blood-oxygen-level–dependent signal changes in specific brain regions. Where available, other neuroimaging parameters such as functional connectivity measures may also be extracted and synthesized. We will conduct a comparison of brain activities between the DP group and the HC group, as well as between the EP group and the HC group. First, we will prepare the text files containing peak coordinates and effect dimensions. For data preprocessing, we will use those files to determine the resulting values of the minimum and maximum potential effect sizes for each voxel. Second, the mean images will be created by averaging the random effects datasets, considering the sample size, study variance, and variations between groups. The effect size maps from various studies will undergo multiple imputations. Third, a family-wise error correction will be used to account for multiple comparisons. If the number of eligible studies permits, we will also conduct subgroup analyses or meta-regression to examine potential moderators (eg, age, sex, imaging parameters).

## Results

The search strategy and screening for the systematic literature review were completed in December 2024 (Multimedia Appendix 2). We identified approximately 30 potentially eligible studies that meet the inclusion criteria during a preliminary scoping search of both English and Chinese databases, suggesting that a quantitative meta-analysis is feasible. In alignment with the objectives of this review, the extracted data will focus on fMRI outcomes associated with TCM syndrome subtypes of MDD. A standardized data extraction form has been developed and is provided in Multimedia Appendices 3 and 4. This form incorporates key elements from fMRI study-reporting criteria, including imaging modality, scanning parameters, analysis pipelines, coordinate systems, and statistical thresholds. These elements aim to ensure consistency and comparability across studies during synthesis. The evidence will be summarized both narratively and quantitatively. Data extraction, quality appraisal, and subsequent data synthesis will begin in September 2025. The review should be completed by December 2025, and the final results will be disseminated through publication in a peer-reviewed journal in 2026.

## Discussion

### Principal Findings

This study protocol proposes a systematic review and meta-analysis to explore the neural mechanisms of TCM-based subtypes of MDD, specifically focusing on DPs and EPs. By applying both qualitative synthesis and quantitative meta-analytic techniques to resting-state fMRI studies, this protocol is designed to assess whether distinct brain activity patterns exist between TCM-defined subtypes. The significant heterogeneity of MDD has historically influenced its diagnosis and therapy [34]. Despite the various classification approaches for MDD, the neurobiological basis of MDD remains poorly understood, and there is still a dearth of compelling evidence for the clinical application value of MDD subtypes in real clinical settings [12]. Although the syndrome-based classification model of TCM (corresponding to the TCM syndrome differentiation and treatment system) is a clinically efficient categorization tool, the connection between this syndrome-based classification model of TCM and the neurological substrate requires further investigation. Our study is the first investigation to comprehensively clarify the neural mechanisms of depression’s neurobiological subtypes from the perspective of TCM, employing both quantitative and qualitative approaches.

### Comparison to Prior Work

Previous studies have focused extensively on Western medicine–based subtyping approaches to MDD, including symptom clusters, genetic polymorphisms, and machine learning–defined neurobiological patterns [35,36]. However, the clinical applicability of these subtypes has remained limited due to replication challenges and inconsistent outcomes [7]. The TCM syndrome classification offers a complementary, theory-driven alternative, with some preliminary studies suggesting that fMRI markers may differ between TCM-defined subtypes [37,38]. This protocol builds upon such observations and aims to systematically synthesize the evidence, which has not yet been undertaken comprehensively.

### Strengths and Limitations

The primary strength of this protocol is its novelty in applying neuroimaging meta-analytic methods to examine brain-based correlations of TCM-defined subtypes of MDD—a field that remains underexplored. In addition, this study follows rigorous methodological frameworks (eg, PRISMA-P, PROSPERO registration) and integrates multiple databases and analytic strategies. Nonetheless, some limitations must be acknowledged. The heterogeneity among the included studies in terms of fMRI parameters, diagnostic criteria, and syndrome classification methods may affect the consistency of the results. The inclusion of non–peer-reviewed literature such as dissertations may also introduce variability in the study quality. Moreover, the current lack of standardized diagnostic criteria for TCM patterns in MDD may challenge cross-study comparability.

### Future Directions

Upon completion, this review is expected to offer a neurobiological perspective on the TCM framework and may help identify reliable imaging biomarkers for MDD subtype differentiation. Future research should aim to develop standardized criteria for TCM syndrome diagnosis that are compatible with neuroimaging methodologies and increase sample sizes through multicenter collaboration. Additionally, further prospective clinical studies could validate whether neuroimaging-defined TCM subtypes predict differential treatment responses.

## Appendix

Multimedia Appendix 1 PRISMA-P checklist.

Multimedia Appendix 2 Search strategy for PubMed.

Multimedia Appendix 3 Methodological challenges in Traditional Chinese Medicine research and functional magnetic resonance imaging data collection and processing.

Multimedia Appendix 4 Study quality rated by using the Agency for Healthcare Research and Quality scale.

## Abbreviations

DP: deficiency pattern
EP: excess pattern
fMRI: functional magnetic resonance imaging
HC: healthy control
MDD: major depressive disorder
MeSH: Medical Subject Headings
PRISMA-P: Preferred Reporting Items for Systematic Reviews and Meta-Analyses Protocols
TCM: Traditional Chinese Medicine
