# Supplementary material for: Neural Activity Disparities in Deficiency and Excess Patterns of Depression: Protocol for a Systematic Review and Meta-Analysis
Source: JMIR Res Protoc. 2025 Sep 18;14:e68996. doi: 10.2196/68996 (PMC12491882; doi:10.2196/68996)
Supplement: Multimedia Appendix 2 [file resprot_v14i1e68996_app2.doc]

**Multimedia Appendix 2.** **The search strategy used in PubMed**

| **Number** | **Search terms** |
| --- | --- |
| **#1** | (all fields) depression  OR  (all fields) depressive disorder  OR  (all fields) MDD  OR  Depressive Disorder, Major[MeSh]  (((depression) OR (depressive disorder)) OR (MDD)) OR (Depressive Disorder, Major[MeSh]) |
| **#2** | (all fields) Chinese Medicine  OR  TCM[tiab]  OR  Medicine, Chinese Traditional[MeSh]  OR  Symptom Complex[MeSh]  ((((Chinese Medicine) OR (TCM[Title/Abstract])) OR (Medicine, Chinese Traditional[MeSh])) OR (Symptom Complex[MeSh]) |
| **#3** | (all fields) Magnetic Resonance Imaging  OR  (all fields) MRI  OR  functional magnetic resonance imaging[tiab]  OR  (all fields) Blood Oxygenation Level Dependent  OR  BOLD[tiab]  OR  ICA[tiab]  OR  ReHo[tiab]  OR  ALFF[tiab]  OR  fALFF[tiab]  OR  (all fields) seed based analysis  OR  (all fields) functional connectivity  OR  (all fields) resting state  OR  (all fields) task-fMRI  OR  (all fields) diffusion MRI  OR  (all fields) DTI  OR  (all fields) DKI  OR  (all fields) magnetic resonance spectroscopy  OR  (all fields) MRS  OR  (all fields) brain connectome  OR  (all fields) brain network  (((((((((((((((((((Magnetic Resonance Imaging) OR (MRI)) OR functional magnetic resonance imaging[Title/Abstract])) OR (Blood Oxygenation Level Dependent)) OR (BOLD[Title/Abstract])) OR (ICA[Title/Abstract])) OR (ReHo[Title/Abstract])) OR (ALFF[Title/Abstract])) OR (fALFF[Title/Abstract])) OR (seed based analysis)) OR (functional connectivity)) OR(resting state)) OR(task-fMRI)) OR(diffusion MRI)) OR(DTI)) OR(DKI)) OR(magnetic resonance spectroscopy)) OR(MRS)) OR(brain connectome)) OR(brain network) |
| **#4** | **#1 AND #2 AND #3** |
