# Supplementary material for: Neural Activity Disparities in Deficiency and Excess Patterns of Depression: Protocol for a Systematic Review and Meta-Analysis
Source: JMIR Res Protoc. 2025 Sep 18;14:e68996. doi: 10.2196/68996 (PMC12491882; doi:10.2196/68996)
Supplement: Multimedia Appendix 3 [file resprot_v14i1e68996_app3.doc]

**Multimedia Appendix 3. Methodological challenges in TCM research and fMRI data collection and processing**

| **Study** | **Clinical study methodologies** | | | | | **fMRI methodologies** | | | | |
| --- | --- | --- | --- | --- | --- | --- | --- | --- | --- | --- |
|  | **TCM**  **diagnosis** | **MDD diagnosis** | **Medication** | **Episodes** | **Severity**  **(mean ± SD)** | **Scanner**  **(sequence; brain coverage** (**mm^2^); FWHM (mm))** | **Subject’s Condition** | **Software** | **Multiple comparison correction** | **Threshold** |
| Zhang et al. 2015 | Two experienced chief TCM physicians | DSM-IV  24-item HAMD≥18 | Washout | NA |  | 3.0T GE; EPI; 220×220; 4 | Eyes closed | SPM8  DPARSF | AlphaSim corrected | p<0.05 corrected |
|  |  |  |  |  |  |  |  |  |  |  |
|  |  |  |  |  |  |  |  |  |  |  |
|  |  |  |  |  |  |  |  |  |  |  |
|  |  |  |  |  |  |  |  |  |  |  |
|  |  |  |  |  |  |  |  |  |  |  |
|  |  |  |  |  |  |  |  |  |  |  |
|  |  |  |  |  |  |  |  |  |  |  |

Abbreviations: Full width at half maximum (FWHM); Statistical Parametric Mapping (SPM); Data Processing Assistant for Resting-State fMRI (DPARSF); Echo-planar imaging (EPI); NA: not available; HAMD: Hamilton Depression Rating Scale; TCM: Traditional Chinese Medicin
