# Supplementary material for: Neural Activity Disparities in Deficiency and Excess Patterns of Depression: Protocol for a Systematic Review and Meta-Analysis
Source: JMIR Res Protoc. 2025 Sep 18;14:e68996. doi: 10.2196/68996 (PMC12491882; doi:10.2196/68996)
Supplement: Multimedia Appendix 4 [file resprot_v14i1e68996_app4.doc]

**Multimedia Appendix 4. Study quality rated by AHRQ scale**

| Study | 1 | 2 | 3 | 4 | 5 | 6 | 7 | 8 | 9 | 10 | 11 | Quality score |
| --- | --- | --- | --- | --- | --- | --- | --- | --- | --- | --- | --- | --- |
| Zhang et al. 2015[1] | Y | Y | N | U | U | Y | Y | Y | N | N | U | 5 |
|  |  |  |  |  |  |  |  |  |  |  |  |  |
|  |  |  |  |  |  |  |  |  |  |  |  |  |
|  |  |  |  |  |  |  |  |  |  |  |  |  |
|  |  |  |  |  |  |  |  |  |  |  |  |  |
|  |  |  |  |  |  |  |  |  |  |  |  |  |
|  |  |  |  |  |  |  |  |  |  |  |  |  |
|  |  |  |  |  |  |  |  |  |  |  |  |  |
|  |  |  |  |  |  |  |  |  |  |  |  |  |
|  |  |  |  |  |  |  |  |  |  |  |  |  |
|  |  |  |  |  |  |  |  |  |  |  |  |  |
|  |  |  |  |  |  |  |  |  |  |  |  |  |
|  |  |  |  |  |  |  |  |  |  |  |  |  |
|  |  |  |  |  |  |  |  |  |  |  |  |  |
|  |  |  |  |  |  |  |  |  |  |  |  |  |
|  |  |  |  |  |  |  |  |  |  |  |  |  |
|  |  |  |  |  |  |  |  |  |  |  |  |  |
|  |  |  |  |  |  |  |  |  |  |  |  |  |

Abbreviations: Y, yes; N, no; U, unclear; AHRQ: Agency for Healthcare Research and Quality; 1. Define the source of information (survey, record review); 2. List inclusion and exclusion criteria for exposed and unexposed subjects (cases and controls) or refer to previous publications; 3. Indicate time period used for identifying patients; 4. Indicate whether or not subjects were consecutive if not population-based; 5. Indicate if evaluators of subjective components of study were masked to other aspects of the status of the participants; 6. Describe any assessments undertaken for quality assurance purposes (e.g., test/retest of primary outcome measurements); 7. Explain any patient exclusions from analysis; 8. Describe how confounding was assessed and/or controlled; 9. If applicable, explain how missing data were handled in the analysis; 10. Summarize patient response rates and completeness of data collection; 11. Clarify what follow-up, if any, was expected and the percentage of patients for which incomplete data or follow-up was obtained

1. Zhang YF, Han Y, Wang YZ, Zhang YF, Jia HX, Jin EH, Deng LG, Li L**: Characterization of resting-state fMRI-derived functional connectivity in patients with deficiency versus excess patterns of major depressi**on*. Complement Ther Me*d 2015**,** 23(1):7-13. doi: 10.1016/j.ctim.2014.12.010.
